# Supplementary material for: Challenges in Lipidomics Biomarker Identification: Avoiding the Pitfalls and Improving Reproducibility
Source: Metabolites. 2024 Aug 19;14(8):461. doi: 10.3390/metabo14080461 (PMC11356033; doi:10.3390/metabo14080461)
Supplement: Supplementary file 1 [file metabolites-14-00461-s001.zip › metabolites-3084482-supplementary.pdf]

Supplementary Material

Table S1: Mass spectrometry settings

Parameters	Full MS	dd-MS2
Resolution	70,000	17,500
Polarity	Positive	Positive
AGC Target	$1e^6$	$5e^5$
Maximum Injection Time (ms)	400	50
Scan Range (m/z)	200 – 1200	200-1200
Loop Count		10
Isolation Window (m/z)		4.0
Collision Energy (normalised to m/z 500, z =1)		30
Minimum AGC Target (for MS/MS triggering)		2.00 e3
Dynamic Exclusion Window (s)		6

See https://sciex.com/tech-notes/life-science-research/metabolomics/untargeted-data-dependent-acquisition--dda--metabolomics-analysi for additional information on standard settings for the ZenoTOF 7600 system in untargeted 'omics analysis

Table S2: MS Dial settings for the PANC1 data

Parameter	Setting
Mass accuracy	MS^1 0.05DA and MS^2 0.025Da tolerance
Maximum charged number	2
Number of threads	7
Minimum peak height	200 amplitude
Mass slice width	0.1Da
Smoothing	Savitzky-Golay filter
Minimum peak width	6 scan
RT tolerance	0.05min
Accurate mass MS^1	0.05Da
Accurate mass MS^2	0.05Da
Identification score cut off	70%
Scoring or filtering for RT	No
Adducts:	$[M+H]^+$, $[M+NH4]^+$, $[M+H-H2O]^+$
Reference file	PANC1 bulk extract 1400 cells/uL
RT tolerance	0.05min
MS^1 tolerance	0.025Da
RT factor	0.5
MS^1 factor	0.5
Peak count filter	10%
Gap filling	No

Table S3: Lipostar settings for the PANC1 data

Parameter	Setting
m/z tolerance (peak detection)	0.05 Da
Maximum charged number	1
Chromatogram filtering threshold	0.97
MS filtering threshold	0.97
Peak exclusion filter (% of base peak)	1 %
Minimum peak height	200 amplitude
Maximum spike size	4 points
Peak extraction half window	1.60 min
S/N filter	3.00

Smoothing	Savitzky-Golay filter
Minimum peak width	6 scan
RT tolerance (alignment)	0.05 min
m/z tolerance (alignment)	0.03 Da
Adducts:	$[M+H]^+$, $[M+NH4]^+$, $[M+H-H2O]^+$
Peak reintegration window	1.00 min
Max adjust time (peak reintegration)	0.10 min
Gap filling	No
MS^1 *m/z* tolerance (identification)	0.05 Da
MS^2 *m/z* tolerance (identification)	0.05 Da

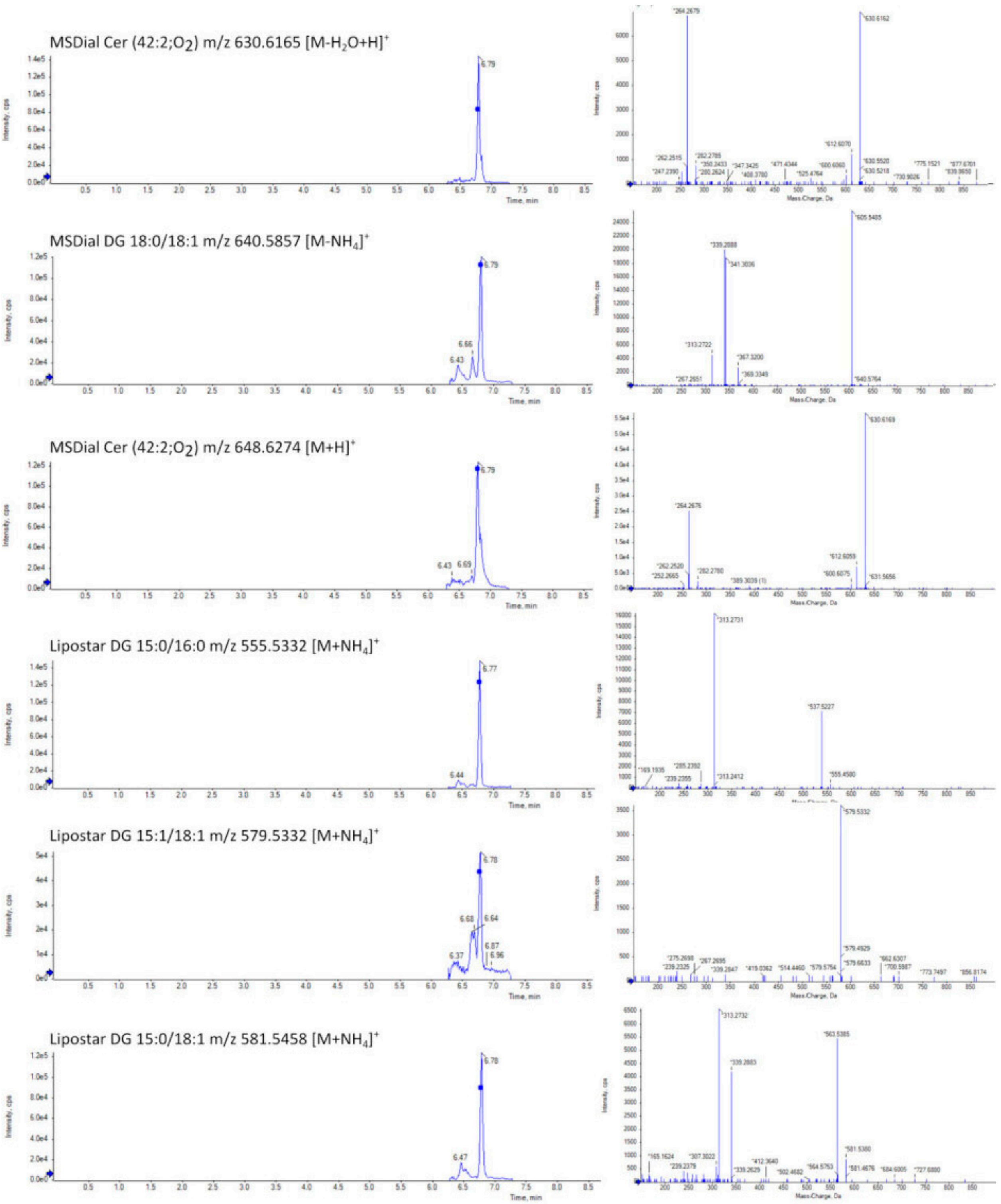

Figure S1: Fragmentation spectra for co-eluting lipids, t_R between 6.78 and 6.80

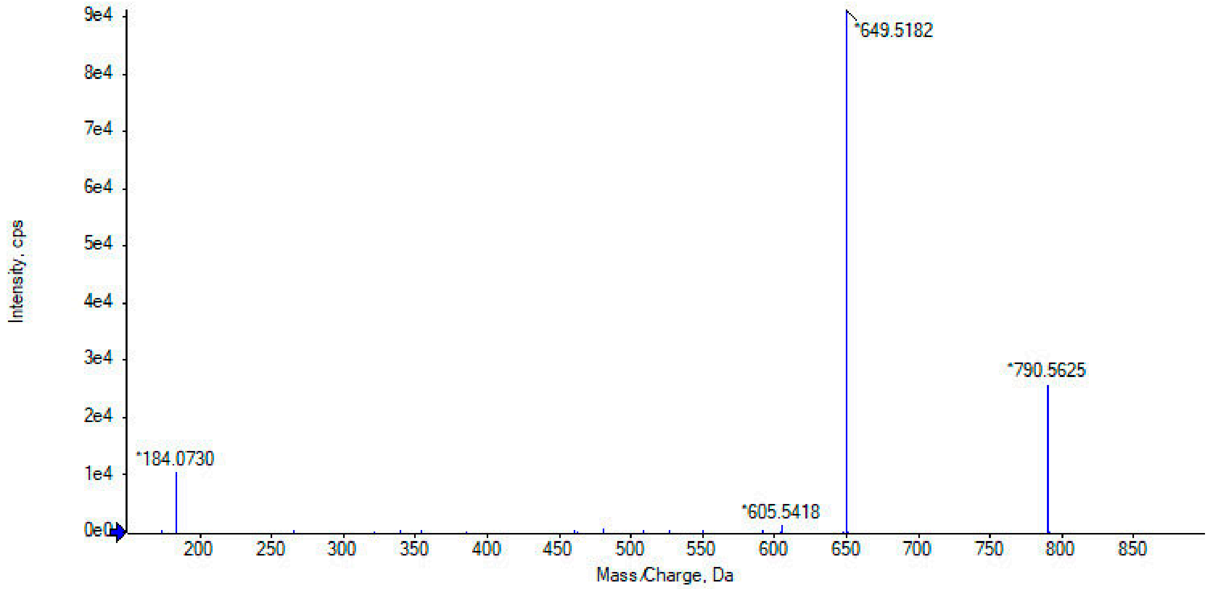

Figure S2: Fragmentation spectra for conflicting identifications contain different indicators of co-eluting PE (neutral loss of 141 Da), PC (fragment ion of 184 Da) and even PS (neutral loss of 185 Da)
